# Urinary Deoxynivalenol Is Correlated with Cereal Intake in Individuals from the United Kingdom

**DOI:** 10.1289/ehp.10663

**Published:** 2007-10-15

**Authors:** Paul C. Turner, Joseph A. Rothwell, Kay L.M. White, YunYun Gong, Janet E. Cade, Christopher P. Wild

**Affiliations:** 1 Molecular Epidemiology Unit and; 2 Nutritional Epidemiology Group, Centre for Epidemiology and Biostatistics, Leeds Institute of Genetics, Health and Therapeutics, University of Leeds, Leeds, United Kingdom

**Keywords:** biomarker, cereal, deoxynivalenol, mycotoxin, U.K., urine

## Abstract

**Background:**

Deoxynivalenol (DON) is a toxic fungal metabolite that frequently contaminates cereal crops. DON is toxic to animals, but the effects on humans are poorly understood, in part because exposure estimates are of limited precision.

**Objectives:**

In this study we used the U.K. adult National Diet and Nutrition Survey to compare 24-hr urinary DON excretion with cereal intake.

**Methods:**

One hundred subjects were identified for each of the following cereal consumption groups: low (mean, 107 g cereal/day; range, 88–125), medium (mean, 179 g/day; range, 162–195) and high (mean, 300 g/day; range, 276–325). DON was analyzed in 24-hr urine samples by liquid chromatography–mass spectrometry after purification on immunoaffinity columns.

**Results:**

DON was detected in 296 of 300 (98.7%) urine samples. Cereal intake was significantly associated with urinary DON (*p* < 0.0005), with the geometric mean urinary levels being 6.55 μg DON/day [95% confidence interval (CI), 5.71–7.53]; 9.63 μg/day (95% CI, 8.39–11.05); and 13.24 μg/day (95% CI, 11.54–15.19) for low-, medium-, and high-intake groups, respectively. In multivariable analysis, wholemeal bread (*p* < 0.0005), white bread (*p* < 0.0005), “other” bread (*p* < 0.0005), buns/cakes (*p* = 0.003), high-fiber breakfast cereal (*p* = 0.016), and pasta (*p* = 0.017) were significantly associated with urinary DON. Wholemeal bread was associated with the greatest percent increase in urinary DON per unit of consumption, but white bread contributed approximately twice as much as wholemeal bread to the urinary DON levels because it was consumed in higher amounts.

**Conclusion:**

The majority of adults in the United Kingdom appear to be exposed to DON, and on the basis of the urinary levels, we estimate that some individuals may exceed the European Union (EU) recommended maximum tolerable daily intake of 1,000 ng DON/kg (bw). This exposure biomarker will be a valuable tool for biomonitoring as part of surveillance strategies and in etiologic studies of DON and human disease risk.

Mycotoxin contamination of cereal crops used for human consumption is common, and those of major concern to health include the aflatoxins, deoxynivalenol (DON), fumonisins, ochra-toxin A, and zearalenone (Miller 1995). The *Fusarium* mycotoxin DON is found primarily on cereal crops of wheat, barley, and maize (corn), and as a result of its stability in processing and cooking (Jackson and Bullerman 1999), levels of human exposure can be high (Canady et al. 2001; Li et al. 1999). DON and other tricothecenes bind to ribosomes, inhibit translation, and activate a signaling pathway known as the ribotoxic stress response (Pestka and Smolinski 2005; Rotter et al. 1996). DON causes acute gastrointestinal effects in animals, including vomiting, feed refusal, and growth retardation (Pestka and Smolinski 2005). There are clear species variations in susceptibility, and this variability may in part reflect the interspecies differences in intestinal detoxification to de-epoxy DON (Yoshizawa et al. 1986), with humans possibly lacking this putative detoxification route (Sundstøl Eriksen and Pettersson 2003).

Epidemiologic studies suggest DON may have played a role in food poisoning incidents in China between 1961 and 1991 (Luo 1994) and in India in 1987 (Bhat et al. 1989) in which many thousands of individuals were affected. DON has potent effects on the immune system, including suppression of the normal immune response to pathogens (Bondy and Pestka 2000; Meky et al. 2001). DON-exposed mice also exhibit kidney pathology similar to IgA nephropathy (Berger disease), the most common type of glomerulonephritis worldwide, which is of unknown etiology (Pestka and Smolinski 2005). In addition, swine exposed to DON show elevated serum IgA, one of the characteristics of IgA nephropathy (Goyarts et al. 2005; Tiemann et al. 2006). A recent review of epidemiologic data highlighted the possibility of DON-induced gastroenteritis, growth faltering, and immunotoxicity in humans, with a possible consequent increase in susceptibility to infectious disease (Pestka and Smolinski 2005). In many regions of the world, chronic exposure to DON is predicted from food contamination data [Canady et al. 2001; Scientific Cooperation (SCOOP) 2003], but to date the health consequences of these exposures remain largely unexamined.

A large collaborative study (SCOOP 2003) assessed the frequency with which predominantly cereal-based foods (44,670 food items) from European Union member states were contaminated by *Fusarium* mycotoxins. Eleven countries in the survey, including the United Kingdom, provided data on the levels of DON on a subtotal of 11,022 food samples. Overall, DON was the most frequently observed mycotoxin, and 57% of all samples tested were contaminated. The report suggested that for some individuals, particularly children, exposure may exceed the maximum tolerable daily intake (TDI) of 1,000 ng/kg (bw), established by the European Commission Scientific Committee for Food (SCF 2002). The report additionally highlighted some of the main limitations in its estimate of DON intake and suggested that the total dietary intake by country should generally be considered an underestimate because of countries not providing data on all food products potentially affected by trichothecene contamination (SCOOP 2003).

Mycotoxin contamination occurs in a range of cereal-containing foods and is heterogeneous within a given food commodity (Cirilloy et al. 2003), making representative sampling difficult. As such, the assessment of mycotoxin exposure at the individual level based on food frequency questionnaires, food diaries, and analysis of DON in food samples can be imprecise. The difficulty of accurate exposure assessment is a major limitation in trying to understand potential associated health risks. In response we have developed a urinary biomarker for DON, and preliminary data suggest that it is sensitive enough to detect human exposure (Meky et al. 2003; Turner et al. 2007). The aim of the present study was to conduct a larger-scale survey to assess the prevalence and level of urinary DON in the U.K. population. Urine samples were available from the U.K. adult National Diet and Nutrition Survey (NDNS) (Henderson et al. 2002), permitting comparison of urinary DON excretion with specific dietary information collected for each individual. Our hypothesis was that a positive correlation would exist between the consumption of cereal products and urinary DON; therefore, individuals were selected for the study following stratification into low, medium, and high cereal intake groups.

## Materials and Methods

### Subject selection from the U.K. adult NDNS

In 2000–2001 the adult NDNS collected 24-hr urine samples from 1,724 individuals 19–64 years of age from across the United Kingdom (Office for National Statistics 2005). Ethics approval was granted both from a Multi-Centre Research Ethics Committee (MREC) and National Health Service Local Research Ethics Committees covering each of the 152 areas from which participants were recruited. All subjects gave written informed consent to participate in the study.

Each individual completed a detailed 7-day weighed food diary. On the basis of dietary records, food groups were identified that provided the major sources of cereal in the U.K. diet; these included breads (wholemeal, white, soft grain, other), breakfast cereals (high-fiber and other), pasta, pizza, fruit pies, biscuits, and buns/cakes. Other potential sources of DON were predicted to provide a smaller contribution and were excluded from the process of selecting individuals for inclusion in the current study. The contribution of cereal from each food group included was determined for each individual over the 7-day period and then divided by seven to create a “total cereal intake” in grams per day. The total cereal intake value was used to rank individuals, and subsequently the 1,724 individuals were divided into deciles. The lowest and highest deciles were excluded to avoid potential outliers. Three groups were created representing low (2nd/3rd decile), medium (5th/6th decile), and high (9th decile) cereal intake. From each of these three groups, 100 individuals were selected on the basis of urine sample availability and that the urine sample had been collected during the period that data in the 7-day diary were provided. Roughly equal numbers of males and female samples were selected for each cereal intake group. Additional NDNS information included age, height, weight, date of sample collection, ethnicity, and vegetarian status.

### Extraction and analysis of urinary DON

Total urinary DON, after hydrolysis of glucuronide conjugates, was measured using an extraction and analysis protocol described in detail elsewhere (Turner et al. 2007). In brief, a 4-mL urine sample was spiked with ^13^C-DON (Biopure Referenzsubstanzen, Tulln, Austria) as an internal standard (IS), to a final concentration of 5 ng/mL urine. The IS was a mixture of isotopes containing predominantly ^13^C_15_-DON [molecular mass (*M*_r_) 311; 81.4%)] but also ^13^C_14_^12^C_1_ DON (*M*_r_ 310; 16.8%). We digested samples for 18 hr at 37°C with 23,000 units of β-glucuronidase (Type IX-A from *Escherichia coli*; Sigma, Dorset, UK)) at pH 6.8. Digested samples were diluted to 16 mL with phosphate-buffered saline (PBS), pH 7.2, and DON isolated using DONtest immunoaffinity columns (Vicam, Watertown, MA, USA) per manufacturer’s instructions. DON was eluted from columns with 4 mL methanol, dried *in vacuo,* and reconstituted in 250 μL of 10% (vol/vol) ethanol for analysis. Two aliquots of a urine sample with known DON levels were also spiked with the internal standard and analyzed with each batch of 20 test samples as a quality control (QC).

DON was analyzed by HPLC (Waters 2795 Separations Module; Waters Corp., Milford, MA, USA) with mass spectrometric detection (Quattro Micro Triple Quadrupole Mass Spectrometer; Micromass UK Ltd., Manchester, UK). Separation of DON was achieved using a Luna C_18_ column (150 × 4.6 mm, 5-μm particle size (Phenomonex, Macclesfield, UK), with isocratic flow (1 mL/min, 20% MeOH, 10 min). Each run also included a wash (75% MeOH, 6 min) and a re-equilibration (20% MeOH, 11 min) phase. Selective ion recording using the combined signal from the most dominant peaks for *a*) DON [major peak Na^+^DON (*m/z* 319.2), and minor peak H+DON (*m/z* 297.2)], and for *b*) the IS, ^13^C-DON [major peak Na^+ 13^C_15_-DON (*m/z* 334.2)], and minor peak Na^+ 13^C_14_^12^C_1_-DON (*m/z* 333.3) were used. External standards of concentrations 10, 20, 50, 100, and 200 ng/mL spiked with internal standard (80 ng/mL) were included at the start of each batch and a further IS-only sample was included at the end. Unknowns and QCs were adjusted for recovery using the IS by reference to a response-ratio calibration curve generated by Quanlynx software (Waters Corp.). For all calibration curves, *R*^2^ was > 0.99. The limit of detection was estimated at 0.1 ng DON/mL of urine, however, the limit of quantification in urine, based on the lowest standard having a coefficient of variation (CV) of < 10%, for this analysis was 0.6 ng DON/mL urine.

### Quality control

Unknown samples were analyzed in 15 batches of 20 samples with two QC samples per batch. The mean level of the QCs was 14.2 ng DON/mL (95% CI, 13.8–14.6; CV 8.0%). Twenty samples from among the 300 urines were re-extracted and analyzed for DON, and a CV of the original and repeat data for each re-run sample obtained. The repeated data were in good agreement with the original set (mean CV, 10.1%; 95% CI, 6.8–13.4). In addition a series of eight samples of buffer only (PBS) were analyzed as blanks. For these blanks a trace peak corresponding to the elution time of DON was observed but quantitatively did not interfere with the analysis (equivalent to < 0.05 ng DON/mL urine).

### Creatinine analysis

Creatinine concentrations were measured in all urine samples using the Bayer clinical method on an ADVIA Chemistry Systems 1650 instrument (Department of Clinical Biochemistry, Leeds General Infirmary, Leeds, UK).

### Statistical analysis

Urinary DON concentrations (nanograms DON per milliliter of urine) were converted to urinary DON amounts (micrograms DON per day) based on 24-hr urine volume. Urinary data were also calculated as nanograms DON per milligram creatinine. Before statistical analysis, DON data were natural log transformed. Urinary DON levels were compared initially with cereal intake as a continuous variable and subsequently to cereal intake groups (low, medium, high). Additional covariates examined included sex, age, body mass index (BMI), date of sample collection, ethnicity, and vegetarian status.

In a second stage of statistical analysis, the individual cereal items (pasta, pizza, wholemeal, white, soft grain, other bread, wholegrain and high-fiber breakfast cereals, other breakfast cereals, biscuits, fruit pies, buns/cakes) used to categorize subjects by cereal intake from the NDNS food diaries were individually compared with log-transformed urinary DON (micrograms DON per day). Covariates associated with the urinary measure (*p* < 0.2) in univariate analysis were retained in the multivariable (MV) model and were considered to contribute significantly to the model if *p* < 0.05. Sex, BMI, and age were retained in all models. The purpose of this analysis was to examine which of the individual cereal food items significantly contributed to urinary DON. Using the regression coefficients from this analysis, the percentage increase in urinary DON per 50 g increase of each specific food item was calculated. The mean consumption for each food item was then used to assess the actual contribution each food was making to urinary DON; again this estimate used the regression coefficients for each food item. Finally, an analysis was made of the levels of urinary DON between consumers and nonconsumers of specific food items. In this last model each food item that as a continuous variable was significantly associated with urinary DON was dichotomized in turn and included in the regression model, with all other food items retained as continuous variables. All data were adjusted for sex, BMI, and age. For ease of presentation, data are back transformed and presented as geometric means with 95% CI. STATA version 7.0 (Stata Corp., College Station, TX, USA) was used for all data analysis.

## Results

Descriptive data for the study group are provided in Table 1. The mean cereal intakes were 107 g cereal/day (range, 88–125), 179 g/day (range, 162–195), and 300 g/day (range, 276–325), for the low, medium, and high groups, respectively. The majority of individuals (188/300; 62.7%) were classified as being overweight (BMI > 25 kg/m^2^), although there was no significant difference in BMI among cereal intake groups (*p* = 0.73). Most individuals (292/300; 97.3%) were white Caucasians. There were a few (18/300, 6.0%) individuals who were vegetarians. Neither ethnicity nor vegetarian status significantly differed between cereal intake groups. There were more females (*n* = 158) than males (*n* = 142) in the study, with a modest trend (*p* = 0.02) toward higher numbers of males and lower numbers of females in the high compared with low cereal intake group. Among consumers of wholemeal bread, males consumed significantly (*p* = 0.002) greater quantities (mean, 53.7 g/day; 95% CI, 45.0–62.4) than females (mean, 34.4 g/day; 95% CI, 26.7–43.0), and males consumed significantly (*p* = 0.005) more white bread (mean, 74.1 g/day; 95% CI, 66.5–81.8) than females (mean, 58.7 g/day; 95% CI, 51.3–66.1). Consumption of other food groups did not differ by sex.

**Figure 1:**
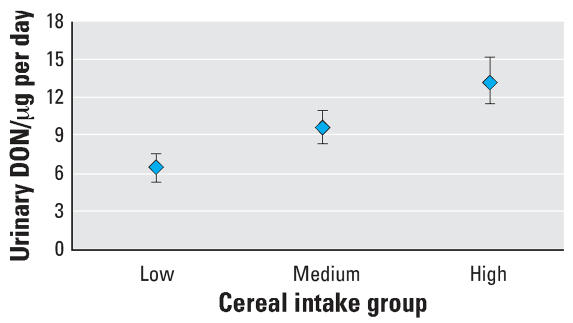
Urinary DON by cereal intake group. Geometric mean and 95% CI values for the level of DON in 24-hr urine samples based on low cereal intake (mean, 107 g; range, 88–125), medium (mean 179; range, 162–195) and high (mean, 300 g; range, 276–325). Data are adjusted for sex, age, and BMI. *p* for trend < 0.0005, adjusted *R*^2^ = 0.182.

DON was detected in 296 of 300 (98.7%) urine samples (geometric mean, 9.42 μg DON/day; range, nondetectable to 65.97 μg/day). Cereal intake, as a continuous variable, was significantly associated with urinary DON (*p* < 0.0005). Within each category of cereal intake individual urinary DON was variable, though there was a significant increase in geometric mean level between the low cereal intake group (6.55 μg/day; 95% CI, 5.71–7.53) and the medium group (9.63 μg/day; 95% CI, 8.39–11.05; *p* = 0.001); between the low and the high groups (13.24 μg/day; 95% CI, 11.54–15.19; *p* < 0.0005); and between the medium- and high-intake groups (*p* = 0.017) after adjustment for sex, BMI, and age (Figure 1). In this model 18.2% of the variance in DON level was explained by the variables considered.

The geometric mean level of urinary DON after adjustment for creatinine was 8.9 ng/mg creatinine (95% CI, 8.2–9.7 ng/mg; range, 0.6–48.2). Urinary DON levels, after creatinine adjustment, were also significantly associated with cereal intake as a continuous variable (*p* < 0.0005) and as a categorical variable (*p* < 0.0005). The geometric mean levels were 7.2 ng/mg (95% CI, 6.2–8.2), 9.2 ng/mg (95% CI, 8.1–10.5), and 10.9 ng/mg (95% CI, 9.5–12.4) for low, medium, and high cereal intake groups respectively.

The interindividual variations in urinary DON were explored further in models assessing the contribution from specific food items. The highest intakes of cereal-based items by weight in descending order were white bread, pasta, wholegrain and high-fiber breakfast cereals, wholemeal bread, other breads and buns/cakes (Table 2). When the amounts of these individual cereals were entered into an MV model with urinary DON as the dependent variable, they explained 23.8% of the variance. Wholemeal bread, white bread, and other bread made the most significant contribution to the interindividual variation in urinary DON (*p* < 0.0005 for each; Table 2. Consumption of fruit pies was negatively associated with urinary DON (*p* = 0.034). Other factors examined in the analysis included, sex, BMI, age, date of sample collection, ethnicity, and vegetarian status. Only sex was significantly (*p* = 0.002) associated with urinary DON, with males having higher levels (geometric mean, 10.78 μg/day; 95% CI, 9.60–12.09) than females (geometric mean, 8.34 μg/day; 95% CI, 7.48–9.32). Other factors were not associated with significant differences in biomarker level.

The percentage increase in urinary DON per 50 g intake of each specific food item was calculated using the regression coefficients from the MV model with the greatest increases associated with wholemeal bread, other bread, buns/cakes, and white bread (Table 2). These data suggest that for an equivalent quantity of food item consumed, wholemeal bread provides the greatest increase in urinary DON. However, the confidence intervals associated with these estimates, based on the confidence intervals of the regression coefficients (data not shown), are quite large. In addition, these food items are not consumed in equal quantities. Analysis based on the mean intake of each specific item therefore provides a better estimate of the contribution of each food to urinary DON. Overall, the mean consumption of white bread was around 3 times greater than that of wholemeal bread, and consumption of this mean quantity of white bread would increase urinary DON by 27.2% (95% CI, 13.4–42.9). This was double the increase associated with the mean intake of wholemeal bread, which provided the second largest overall contribution (Table 2), although again, the CI values for these estimates were relatively large.

**Figure 2:**
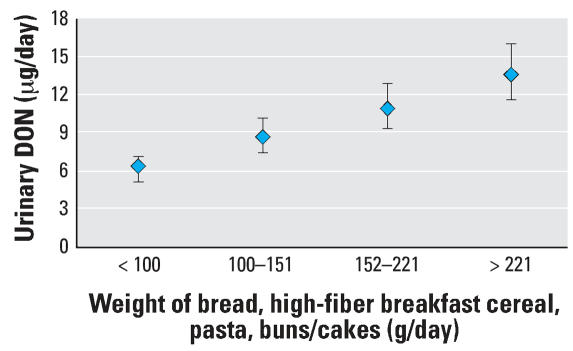
Urinary DON by intake of specific food items (total bread, high-fiber breakfast cereal, pasta, buns/cakes). Geometric mean and 95% CI are shown for the level of DON in 24-hr urine samples based on consumption specific foods. All data are adjusted for sex, age, and BMI. *p* < 0.0005, adjusted *R*^2^ = 0.238.

The consumption, in grams per day, of those food items positively associated with urinary DON (all breads, pasta, high-fiber breakfast cereal, buns/cakes) was combined. The mean weight of consumption of these combined specific foods was 143 g/day range (19–304 g/day). Individuals were categorized into quartiles of amounts consumed (*n* = 75 per group), with those in the lowest group consuming < 100 g/day, and those in the highest group consuming > 221 g/day. From the lowest to the highest intake groups, there was a greater than 2-fold mean increase in urinary DON (*p* < 0.0005), geometric mean, 6.14 μg/day (95% CI, 5.17–7.12) to 13.63 μg/day (95% CI, 11.61–15.99) for low and high quartiles, respectively (Figure 2).

Using the specific food items associated with urinary DON, we dichotomized individuals into consumers or nonconsumers of these items. Consumers of wholemeal bread and high-fiber breakfast cereal had significantly higher levels of urinary DON compared with nonconsumers (Table 3). A modest increase in urinary DON was observed for consumers of other bread, whereas consumers of buns/cakes, white bread, and pasta showed nonsignificant trends to higher urinary DON.

## Discussion

DON is a frequent contaminant of cereal crops throughout the world, including Europe (SCOOP 2003), and has a variety of toxicities predominantly targeting the gastrointestinal and immune systems (Bondy and Pestka 2000; Meky et al. 2001; Pestka and Smolinski 2005). The evidence from animal studies clearly identifies DON as a potent toxin, albeit with varying susceptibility among species (Pestka and Smolinski 2005); ecologic data from China and India suggest a link between exposure and some human poisoning outbreaks, but the dose–response relationships are inconclusive (Bhat et al. 1989; Luo 1994). This uncertainty as to the susceptibility of humans to DON and the unknown effect of environmental levels of exposure on human health therefore implies the need for improved epidemiologic data. In this context, the absence of a validated DON exposure biomarker continues to hamper reliable and accurate assessment of intake at the individual level. To address this we have developed a robust assay to measure total urinary DON after β-glucuronidase treatment of urine to deconjugate DON–glucuronide and have previously reported preliminary results in a small number of Chinese and U.K. subjects (Meky et al. 2003; Turner et al. 2007).

The adult NDNS provided a comprehensive assessment of dietary intake over a 1-week period for a cross-section of U.K. adults. The availability of urine samples from these individuals during the collection of detailed food diaries therefore provides an opportunity to investigate the association between diet and urinary DON. DON was detected in 296 of 300 (98.7%) of the urine samples, with a positive association being observed with cereal consumption (Figure 1). In this larger survey of 24-hr urines, urinary DON concentration after creatinine adjustment (geometric mean, 8.9 ng/mg; 95% CI, 8.2–9.7; *n* = 300) was similar to those previously reported by Turner et al. (2007) in our preliminary study in the United Kingdom using first-morning void urines (geometric mean, 7.2 ng/mg; 95% CI, 4.9–10.5; *n* = 25). In an earlier study by Meky et al. (2003), using a slightly different methodology we reported mean levels of urinary DON in China of 30 ng DON/mL (range, 4–94 ng/mL; *n* = 15), and these are similar to the levels in the data from the current study when unadjusted for urine volume or creatinine (mean, 7.5 ng DON/mL; range, non-detectable to 56.4 ng/mL; *n* = 300). For many studies of environmental exposure, only spot urine samples are available and toxicant concentrations are then adjusted for creatinine level. Advocates suggest that this adjustment provides a better estimate of intake and a tighter correlation with toxicant levels in other biofluids (Hill et al. 1995; Shealy et al. 1997; To-Figueras et al. 1997). However, a number of external factors can influence creatinine levels [reviewed by Boeniger et al. (1993)], and thus 24-hr urines are often regarded as the gold standard (Barr et al. 2005). It is of note that after adjustment for creatinine in our study, urinary DON was still significantly associated with cereal intake (*p* < 0.0005).

When the individual food groups used to categorize cereal intake were investigated as continuous variables, wholemeal bread, other bread, white bread, buns/cakes, high-fiber breakfast cereal, and pasta were found to significantly contribute to the interindividual variation in urinary DON levels. For these food items positively associated with the biomarker, the amounts consumed were combined, and total intake of cereals from these sources was compared with urinary DON. This latter model provided a slightly improved explanation of the variance in urinary DON compared with the broader measure of cereal intake. The explanation for the modest negative association observed between fruit pie consumption and urinary DON is unclear, but in any case consumption was associated with < 3% reduction in urinary DON (Table 2).

The foods groups associated with urinary DON were predominantly wheat based, particularly the three main bread groups. When individuals were subsequently dichotomized into consumers and nonconsumers of specific food items, consumption of wholemeal bread showed the strongest association with urinary DON. Consumers of high-fiber breakfast cereal also had significantly higher urinary DON levels compared with those of nonconsumers. Of those food items associated with urinary DON, wholemeal bread provided the greatest percent increase in urinary DON per unit of consumption, which is consistent with the fact that DON contamination of wheat tends to be greatest in the bran fraction (Joint Expert Committee on Food Additives 2001). However, the intake of white bread provided the largest contribution to cereal intake because of the frequent and higher-level consumption, with only 8% of subjects being nonconsumers. Consequently, the major contribution to total urinary DON appears to be from bread with white bread overall, providing around double the contribution of wholemeal bread. This finding is in good agreement with SCOOP (2003), which predicted that bread and high-fiber breakfast cereals were the most likely sources of DON exposure for U.K. adults. In the MV model, sex was also significantly associated with urinary DON (*p* = 0.002), with males having an approximately 23% higher level than females, a difference that remained after adjustment for cereal intake or specific cereal-based food intake. The reason for this difference by sex is unknown, although it is not explained by difference in body weight.

A crude estimate of daily DON intake was made based on *a*) the total amount of urinary DON; *b*) an assumption that 50% of the ingested DON was being excreted in the urine [based on Goyarts and Dänicke (2006) and Meky et al. (2003)]; and *c*) the urinary DON originated from DON intake in the previous 24 hr. Accordingly, for each individual the intake was calculated on the basis of the urinary DON level and individual body weight. For the 300 individuals the mean intake was calculated as 319 ng/kg(bw)/day. A recommended TDI for DON ingestion was set at 1,000 ng/kg (bw) (SCF 2002); it is notable that 4 of 300 individuals (1.3%) would be predicted to exceed the TDI using the above calculation and 3 of these 4 people were from the high cereal intake group. Previous estimates of DON exposures in the United Kingdom were of the same order of magnitude as those derived from our urinary biomarker data: specifically, adult male and female intakes were estimated as 176 and 142 ng/kg (bw)/day, although it was noted that these may be underestimates due to countries not providing data on all food products potentially affected by trichothecene contamination (SCOOP 2003).

The high-intake group in this study comprises individuals from the 9th decile of cereal intake, whereas subjects in the highest decile (10th decile) were not examined. Individuals from this latter group would be expected to have still higher DON ingestion. Thus it is not unreasonable based on our data to estimate that some 5% of the U.K. adult population may exceed the TDI for DON intake. These values, however, should be interpreted with caution, partly because of the uncertainty in the percentage transfer of DON into human urine and the fact that the kinetics of human urinary excretion of DON and its metabolites has not been established. DON itself is a water-soluble mycotoxin, and therefore the majority is likely to be excreted relatively rapidly, as is observed for other mycotoxins in humans (Groopman et al. 1992). The DON–glucuronide appears to be formed rapidly in swine and to have serum clearance kinetics similar to that of the parent compound (Goyarts and Dänicke 2006).

In this study urinary DON levels were compared with dietary intake over a 7-day period. The fact that DON excretion is likely to be rapid means that an average cereal intake over a week may not be as closely related to urinary DON as would be intakes over the previous 24–48 hr. In addition, mycotoxin contamination of cereals will be heterogeneous, and we did not have a measure of this in our study. Finally, not all potential sources of DON were accounted for in the stratification of subjects, including polenta and cereal-based snacks. Each of these limitations may in part explain why the MV model for food intake accounted for only 23.8% of the variance in urinary DON.

In summary, this study indicates that DON exposure is ubiquitous in the United Kingdom and urinary levels of DON are positively correlated with cereal intake, particularly bread consumption. The variation in urinary DON among individuals with similar total cereal intakes highlights the need for specific biomarkers in order to assess more precisely exposure at the individual level. The availability of this biomarker will assist in bio-monitoring at the population level and provide a tool for application in etiologic studies to assess the potential risks associated with this widespread exposure to DON.

## Figures and Tables

**Table 1:** Descriptive data of selected individuals by cereal intake group.

		Cereal intake group			
	Total (*n* = 300)	Low (*n* = 100)	Medium (*n* = 100)	High (*n* = 100)	*p*-Value for trend
Cereal intake (g/day)^a^	196 (88–325)	107 (88–125)	179 (162–195)	300 (276–325)	< 0.0005
Sex					0.02
Female	158	62	50	46	
Male	142	38	50	54	
Age^a^ (years)	42.9 (19–64)	44.1 (19–64)	40.9 (19–64)	43.7 (19–64)	0.81
BMI^b^ (kg/m^2^)	27.1 (26.4–27.7)	27.4 (26.3–28.5)	26.8 (25.6–27.8)	27.1 (26.0–28.2)	0.73
Ethnicity^c^	292/300	96/100	97/100	99/100	0.19
Vegetarian	18/300	4/100	6/100	8/100	0.23

a Mean (range).

b Mean (95% CI).

c Number of individuals that were white Caucasians.

**Table 2:** Effect of intake of specific food items on urinary DON.

Food item	Intake [g/day mean (range)]	Regression coefficient	*p*-Value^a^	Percentage increase in urinary DON per 50 g food/day^b^ (95% CI)	Overall percentage contribution to urinary DON increase^c^ (95% CI)
Pasta	26 (0–179)	0.0027	0.017	14.3 (2.4 to 27.6)	7.2 (1.3 to 13.5)
White bread	61 (0–243)	0.0040	< 0.0005	21.8 (10.8 to 134.0)	27.2 (13.4 to 42.9)
Wholemeal bread	18 (0–183)	0.0070	< 0.0005	41.8 (23.6 to 62.7)	13.4 (7.9 to 19.2)
Other bread	18 (0–196)	0.0061	< 0.0005	35.5 (17.9 to 55.6)	11.5 (6.1 to 17.2)
HF breakfast cereal	21 (0–194)	0.0031	0.016	16.8 (3.0 to 3.2)	11.5 (6.1 to 17.2)
Fruit pies	3 (0–71)	–0.0087	0.034	–35.3 (–56.2 to –3.2)	–2.6 (–4.9 to –0.2)
Buns/cakes	18 (0–155)	0.0054	0.003	31.2 (10.1 to 56.4)	10.2 (3.5 to 17.5)

HF, high fiber.

a Multiple linear regression of intake of food item as a continuous variable against urinary DON level. All models were adjusted for sex, BMI ,and age.

b 95% CI, 95%confidence intervals based on the confidence intervals of the regression coefficients (values not shown).

c The contribution of the mean intake of the specific food items (column 2 of this table, “Intake g/day”) to the increase in urinary DON.

**Table 3:** Effect of being a consumer or nonconsumer of specific food items on urinary DON.

Food item	No. of nonconsumers (%)	Urinary DON in nonconsumers [μg/day mean (95% CI)]	Urinary DON in consumers [μg /day mean (95% CI)]	*p*-Value^a^
Pasta	142 (47)	8.78 (7.86–9.90)	10.00 (8.96–11.16)	0.126
White bread	23 (8)	8.78 (6.50–11.97)	9.51 (8.69–10.30)	0.655
Wholemeal bread	180 (60)	7.79 (7.04–8.68)	12.46 (10.94–14.19)	< 0.0005
Other bread	146 (49)	8.69 (7.71–9.70)	10.20 (9.05–11.38)	0.05
HF breakfast cereal	150 (50)	8.35 (7.48–9.32)	10.61 (9.51–11.85)	0.005
Fruit pies	266 (89)	9.60 (8.95–10.51)	7.56 (6.00–9.60)	0.056
Buns/cakes	109 (36)	8.60 (7.56–9.80)	9.90 (8.96–11.16)	0.067

HF, high fiber.

a Multiple linear regression, with adjustment for other food items as continuous variables. All models were adjusted for sex, BMI, and age.
